# The Prognostic Value of Tricuspid Annular Dimensions in TAVI Patients: A CT-Based Retrospective Analysis of Risk Stratification and Long-Term Outcomes

**DOI:** 10.3390/jcm14093191

**Published:** 2025-05-05

**Authors:** Nikolaos Schörghofer, Christoph Knapitsch, Gretha Hecke, Nikolaus Clodi, Lucas Brandstetter, Matthias Hammerer, Klaus Hergan, Uta C. Hoppe, Elke Boxhammer, Bernhard Scharinger

**Affiliations:** 1 Department of Radiology, Paracelsus Medical University of Salzburg, 5020 Salzburg, Austria; 2 Department of Internal Medicine II, Division of Cardiology, Paracelsus Medical University of Salzburg, 5020 Salzburg, Austria; m.hammerer@salk.at (M.H.);

**Keywords:** aortic valve stenosis, TAVI, computed tomography, tricuspid annular dimensions

## Abstract

**Background**: Transcatheter aortic valve implantation (TAVI) has transformed the treatment of severe aortic stenosis (AS), particularly in high-risk patients. However, comorbidities such as pulmonary hypertension (PH) and secondary tricuspid regurgitation (TR) contribute to adverse outcomes. Tricuspid annulus (TA) dilatation (TAD), a key marker of right ventricular dysfunction, has been associated with PH and TR progression. While echocardiographic assessment of TA has limitations, cardiac computed tomography (CT), routinely performed before TAVI, enables precise TA measurement. This study aimed to determine clinically relevant TA and TA indexed to body surface area (TA/BSA) cut-offs and assess their prognostic significance for long-term mortality. **Methods**: This retrospective, single-center study included 522 patients who underwent transfemoral TAVI between 2016 and 2022. Pre-procedural CT-derived TA measurements were analyzed to establish cut-off values predictive of right ventricular dysfunction in TAVI. Receiver operating characteristic (ROC) analysis was performed, and Kaplan–Meier survival curves, log-rank tests, and Cox regression were used to assess the impact of TA dimensions on long-term survival. **Results**: TAD correlated moderately with right ventricular dysfunction, with optimal cut-offs identified as TA ≥ 44.50 mm and TA/BSA ≥ 23.00 mm/m^2^. However, Kaplan–Meier and Cox regression analyses demonstrated no significant association between TA or TA/BSA and long-term survival, with area under the curve (AUC) values close to 0.50, indicating poor prognostic value. **Conclusions**: Despite its relevance regarding right ventricular dysfunction in TAVI patients, TAD does not independently predict long-term mortality following TAVI. These findings challenge prior assumptions and suggest that TA dimensions alone should not guide risk stratification in TAVI patients. Further research is needed to refine prognostic models integrating multiple clinical and imaging parameters.

## 1. Introduction

Transcatheter aortic valve implantation (TAVI) has revolutionized the treatment of symptomatic severe aortic stenosis (AS), offering a minimally invasive alternative to surgical valve replacement [1,2]. Initially reserved for high-risk patients, its success has rapidly expanded its indications, with recent trials demonstrating favorable outcomes even in younger, lower-risk populations [3,4,5,6]. Yet, despite these advancements, patient prognosis often remains influenced by comorbidities with pulmonary hypertension (PH) being one of the most relevant [7,8], by potentially complicating the course of AS and impacting post-TAVI outcomes.

PH is not merely an incidental finding but a key driver of right ventricular (RV) dysfunction and structural remodeling. It promotes tricuspid annulus (TA) dilatation (TAD), setting the stage for secondary tricuspid regurgitation (TR)—a condition that further increases right heart strain and has been linked to increased morbidity and mortality. Despite its clinical significance, secondary TR is often overlooked until it reaches an advanced stage, underscoring the urgent need for early recognition and intervention [9,10,11].

Echocardiographic assessment of the TA has considerable limitations, particularly in accurately quantifying annular dilatation [12]. However, computed tomography angiography (CTA), routinely performed as part of pre-TAVI planning, provides a unique opportunity to obtain high-resolution, three-dimensional measurements of the TA [13]. This advanced imaging technique allows for the precise quantification of TAD, potentially transforming risk assessment and guiding clinical decision-making in patients with PH and TR undergoing TAVI.

Given the prognostic implications of TAD in the current literature [14] and its intricate relationship with PH and TR, identifying potentially clinically meaningful cut-off values for TA and TA indexed to body surface area (TA/BSA) is of great interest to the cardiologist [15]. The objective of this study was to leverage CTA-derived TA measurements in a large cohort of TAVI patients to establish optimal thresholds for risk stratification and to evaluate their association with long-term mortality. By bridging the gap between advanced imaging and clinical outcomes, this research aims to improve patient selection, assessment of prognosis, and ultimately the management of patients with severe AS undergoing TAVI.

## 2. Material and Method

### 2.1. Study Population

This retrospective, single-center study analyzed data from patients who underwent TAVI at Paracelsus Medical University Hospital Salzburg between January 2016 and June 2022. Initially, the study cohort comprised 585 patients. However, 63 were excluded due to incomplete CT data or the absence of an end-diastolic phase necessary for precise measurement of the septolateral TA diameter. Consequently, the final analysis included 522 patients.

### 2.2. Ethics Declaration

The study was approved by the Ethics Committee of the state of Salzburg, Austria (EK-Nr. 1082/2024). All data collection and management adhered to ethical principles outlined in the Declaration of Helsinki and the ICH-GCP (International Council for Harmonisation of Technical Requirements for Pharmaceuticals for Human Use—Good Clinical Practice) guidelines. Given the retrospective nature of the study, the requirement for written informed consent was waived by the State of Salzburg Ethics Commission.

### 2.3. Data Extraction

Patient data were retrieved from the ORBIS electronic medical records platform (Agfa Healthcare, Version 08043301.04110DACHL) and the Uniklinikum Salzburg medical archiving system (Krankengeschichtsarchiv System, Softworx by Andreas Schwab TM, 2008). Extracted information included patient medical records, admission and discharge documentation, and echocardiographic and imaging reports associated with the hospitalization for the TAVI procedure.

### 2.4. Transthoracic Echocardiography

Transthoracic Echocardiography (TTE) was routinely performed approximately 1–2 months before the TAVI procedure using either an iE33 or Epiq 5 ultrasound system (Philips Healthcare, Hamburg, Germany). Examinations were conducted by experienced clinicians with at least four years of specialized training in echocardiography. The assessment of AS severity was based on current European Society of Cardiology (ESC) and American College of Cardiology/American Heart Association (ACC/AHA) guidelines [16]. Left ventricular ejection fraction (LVEF) was measured using Simpson’s biplane method.

To estimate systolic pulmonary artery pressure (sPAP), systolic tricuspid regurgitation velocity (TRVmax) was assessed using continuous wave Doppler imaging across the tricuspid valve. The maximum velocity of the tricuspid regurgitant jet (TRVmax) was used to derive right atrioventricular pressure gradient using the simplified Bernoulli equation (4 × TRVmax^2^). Right atrial pressure (RAP) was estimated based on inferior vena cava (IVC) diameter and its collapsibility during inspiration, reflecting central venous pressure [17]. The sPAP was then calculated as follows: sPAP = 4 × TRVmax^2^ + RAP.

PH was identified according to ESC guidelines using the following criteria: sPAP ≥ 40 mmHg, TRVmax ≥ 2.8 m/s, and a TAPSE/sPAP ratio < 0.55 mm/mmHg [18].

TR was assessed using color Doppler imaging to visualize the regurgitant jet and quantify its severity. Additionally, continuous wave Doppler was employed to measure TRVmax, and pulsed wave Doppler was used to detect hepatic vein flow reversal as a secondary indicator of significant regurgitation. TR severity was classified based on ESC guidelines [19], considering parameters such as vena contracta width, jet area relative to right atrial size, and hepatic vein flow characteristics.

### 2.5. Computed Tomography Angiography Image Acquisition, Image Analysis, and Measurement of Tricuspid Annulus Diameters

Patients included in this study routinely underwent pre-interventional, ECG-gated CT) covering the region from the apex of the lungs to the proximal femoral arteries. These scans were performed to obtain the necessary measurements for TAVI. CTA examinations were conducted using either a 256-slice or a 128-slice dual-source CT scanner (Revolution, General Electric Healthcare, IL, USA or Somatom Definition AS+, Siemens Healthcare, Erlangen, Germany). The tube voltage was adjusted according to patient size (80–120 kVp), and active tube current modulation was applied. A bolus-tracking technique was used for contrast enhancement, involving the injection of 100 mL of a non-ionic iodinated contrast agent, followed by 70 mL of saline solution, at a flow rate of 3.5–5 mL/s.

**Figure 1:**
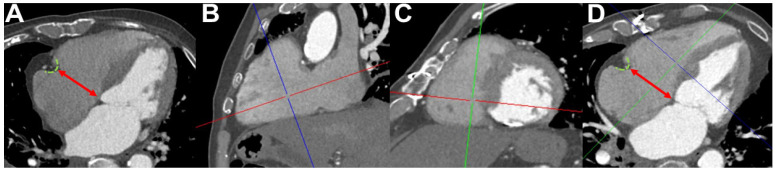
Measurement of tricuspid annular diameter on strictly axial slices and on manually angulated slices: First, the right atrioventricular groove was identified (green dashed lines in (**A**,**D**)). Then, the tricuspid annular diameter (red double-headed arrow) was measured on strictly axial slices by drawing a straight line orthogonal to the long axis of the ventricle, directed toward the interventricular septum (**A**). Next, the axes were manually angulated (**B**,**C**) to obtain a four-chamber view (**D**), and the same measurement was repeated under these angulated conditions.

TA diameter was measured by a radiologist with subspecialization in cardiovascular imaging using a dedicated workstation (Impax, Agfa-Gevaert, Mortsel, Belgium). First, measurements were performed on strictly axial slices from a single CTA image. The largest distance between the right atrioventricular groove to the interventricular septum was recorded as the septo-lateral TA diameter in mm as described previously [20] (Figure 1A).

To investigate whether patient-specific variations in the cardiac axis, and, therefore, the oblique orientation of the TA, might lead to different measurement results than those obtained from strictly axial slices, a subset of 30 patients was randomly selected for additional, manually angulated four-chamber view measurements (Figure 1B–D) as described previously [14]. The same radiologist, blinded to the initial measurement results, performed these repeat assessments.

### 2.6. TAVI Procedure

All patients underwent transfemoral TAVI using second- or third-generation valve systems, specifically the CoreValve™ Evolut™ R and CoreValve™ Evolut™ Pro (Medtronic Inc., Minneapolis, MN, USA), following established procedural protocols. Pre-procedural imaging, including TTE, CTA, and, when indicated, transesophageal echocardiography (TEE) was used to confirm diagnosis and guide procedure planning, particularly valve size selection.

### 2.7. Outcomes

The primary outcome of this study was long-term overall survival, which was tracked from the date of the TAVI procedure up to a maximum follow-up period of 84 months.

### 2.8. Statistical Analysis

To assess the reliability of the TA measurements, the intraclass correlation coefficient (ICC) was calculated using R (Version 4.2.3, R Foundation for Statistical Computing, Vienna, Austria). The ICC was determined for the agreement between the uncorrected and axis-corrected measurements of TA in 30 patients. A two-way mixed-effects model with absolute agreement was used, as implemented in the irr package. ICC values were interpreted based on established guidelines, with values below 0.50 indicating poor reliability, between 0.50 and 0.75 moderate reliability, between 0.75 and 0.90 good reliability, and above 0.90 excellent reliability.

Further data analysis was conducted using IBM SPSS Statistics version 25 (Armonk, NY, USA). Normality was evaluated using the Kolmogorov–Smirnov test and visually inspected with Q-Q plots. Normally distributed data were expressed as mean ± standard deviation (SD), while non-normally distributed data were reported as median ± interquartile range (IQR). Categorical data were presented as absolute numbers and percentages. Comparisons of normally distributed data were performed using Student’s *t*-test, while the Mann–Whitney U-test was used for non-parametric variables. Categorical variables were analyzed using the χ^2^ test.

Cut-off values for TA in the overall cohort were determined using receiver operating characteristic (ROC) curve analysis, with area under the curve (AUC) and Youden Index (YI) calculations. These analyses were conducted for TAPSE/sPAP ratio < 0.55 mm/mmHg. The radiological cut-off values identified in the AUROC analyses were utilized to construct Kaplan–Meier survival curves and perform corresponding log-rank tests. Additionally, univariate and multivariate Cox-regression analysis were calculated. Finally, AUC values were calculated to demonstrate the prediction of TA and TA/BSA regarding mid- and long-term mortality in TAVI patients.

Statistical significance was defined as *p* ≤ 0.050.

## 3. Results

### 3.1. Study Cohort and Baseline Characteristic

The study cohort consisted of 522 patients, with a nearly equal distribution of sexes (males: 49.8%). The mean age was 82.1 ± 5.2 years, with 71.5% being older than 80. Arterial hypertension was highly prevalent (86.8%), followed by coronary heart disease (50.6%) and atrial fibrillation (36.2%). Diabetes mellitus was present in 27.0% of patients, while chronic obstructive pulmonary disease (11.3%) and peripheral arterial disease (8.4%) were less common.

Regarding cardiac function, the mean left ventricular ejection fraction was 53.0 ± 9.8%, and the mean aortic valve peak velocity was 4.4 ± 0.6 m/s, reflecting significant aortic stenosis. Pulmonary hypertension was common, with 44.1% of patients having sPAP ≥ 40 mmHg. RV dysfunction markers included a mean TAPSE of 21.9 ± 4.7 mm and a TAPSE/sPAP ratio of 0.8 ± 0.8. TAD was notable, with a mean annular diameter of 40.4 ± 6.7 mm, corresponding to a TA/BSA of 22.4 ± 4.2 mm/m^2^. Further baseline characteristic details can be found in Table 1.

### 3.2. ICC

ICC analysis was conducted to evaluate the agreement between axis-corrected and non-corrected tricuspid annular diameter measurements in 30 patients. Using a two-way model for consistency (ICC [C,1]), the analysis yielded an ICC of 0.915 (95% CI: 0.829–0.959), indicating excellent reliability. Given the strong agreement between the two measurement methods, we opted for the examiner-friendly approach of non-axis-corrected measurements for further analyses.

### 3.3. Cut-Off Values of TA Dimensions for Right Ventricular Dysfunction

**Figure 2:**
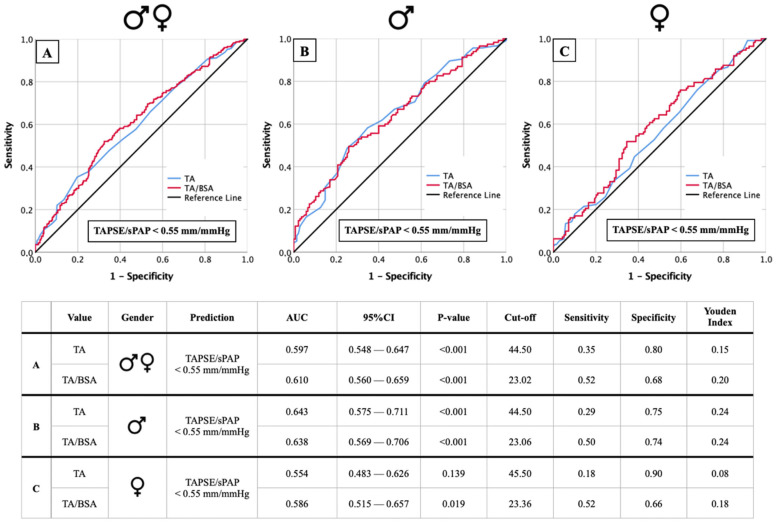
AUC analyses of TA and TA/BSA for prediction of TAPSE/sPAP < 0.55 mm/mmHg with concerning cut-off values, Youden Index, sensitivity, and specificity for the whole cohort (**A**), male (**B**) and female subgroups (**C**).

Figure 2 illustrates the predictive value of tricuspid annulus (TA) dimensions and TA indexed to body surface area (TA/BSA) for identifying right ventricular dysfunction, as defined by a TAPSE/sPAP ratio of <0.55 mm/mmHg. In the overall cohort, the analysis demonstrates a modest discriminatory ability, with AUC values ranging from 0.554 to 0.643. The optimal cut-off values for TA were found to be 44.50 mm in most cases, except for one subgroup where it was slightly higher at 45.50 mm, while TA/BSA thresholds ranged from 23.02 to 23.36 mm/m^2^. Sensitivity remained relatively low across the analyses, reaching a maximum of 0.52, whereas specificity was consistently higher, peaking at 0.90. When considering sex-specific differences, the predictive performance varied slightly, though the general trends remained similar. The results highlight that while TA dimensions play a role in identifying patients with impaired right ventricular function, their ability to serve as robust independent markers is limited.

### 3.4. Comparison of Clinical Characteristics According to TA and TA/BSA Subgroups

Table 2 highlights significant differences in clinical characteristics based on TA dimensions (cut-off: 44 mm) and TA/BSA (cut-off: 23.00 mm/m^2^), both of which were derived from the TAPSE/sPAP stratification. Patients with a larger TA (≥44 mm) and TA/BSA (≥23.00 mm/m^2^) exhibited clear distinctions in pulmonary hypertension markers and demographic factors.

Sex distribution showed a strong association with TA size, as men were significantly more prevalent in the TA ≥ 44 mm group (65.8% vs. 42.7%, *p* < 0.001), whereas in the TA/BSA stratification, men were more frequent in the smaller TA/BSA group (52.4% vs. 44.6%, *p* = 0.041). Age differences were particularly pronounced in the TA/BSA stratification, with 80.8% of patients in the TA/BSA ≥ 23.00 mm/m^2^ group being older than 80 years, compared to 65.1% in the smaller TA/BSA cohort (*p* < 0.001).

Markers of pulmonary hypertension were significantly elevated in patients with larger TA and TA/BSA. A TRVmax ≥ 2.8 m/s was more common in the TA ≥ 44 mm group (56.5% vs. 40.7%, *p* = 0.001) and the TA/BSA ≥ 23.00 mm/m^2^ group (54.0% vs. 39.7%, *p* = 0.003). Similarly, sPAP was higher in patients with larger TA (43.3 ± 16.8 mmHg vs. 36.4 ± 16.7 mmHg, *p* < 0.001) and larger TA/BSA (42.7 ± 16.1 mmHg vs. 36.1 ± 17.1 mmHg, *p* < 0.001).

Although LVEF remained similar between groups (TA: 51.8 ± 10.8% vs. 53.6 ± 9.3%, *p* = 0.069; TA/BSA: 52.2 ± 10.1% vs. 53.6 ± 9.6%, *p* = 0.125), left ventricular end-diastolic diameter (LVEDD) was slightly higher in patients with larger TA (47.6 ± 6.7 mm vs. 45.8 ± 6.7 mm, *p* = 0.012).

### 3.5. Impact of TA Dimensions on Short-Term Outcomes

Short-term outcomes, including procedural and 30-day complications, were analyzed in relation to TA and TA/BSA dimensions (Table 3).

Procedural mortality was rare (0.6% in both TA groups), and 30-day mortality remained below 2% across all subgroups. Stroke or TIA occurred in 2–3% of patients, while pacemaker implantation was required in approximately 12–13%. Major vascular complications were similarly infrequent, with rates below 5% in all groups. Overall, larger annular dimensions were not associated with increased short-term risk.

### 3.6. Impact of TA Dimensions on Long-Term Survival

**Figure 3:**
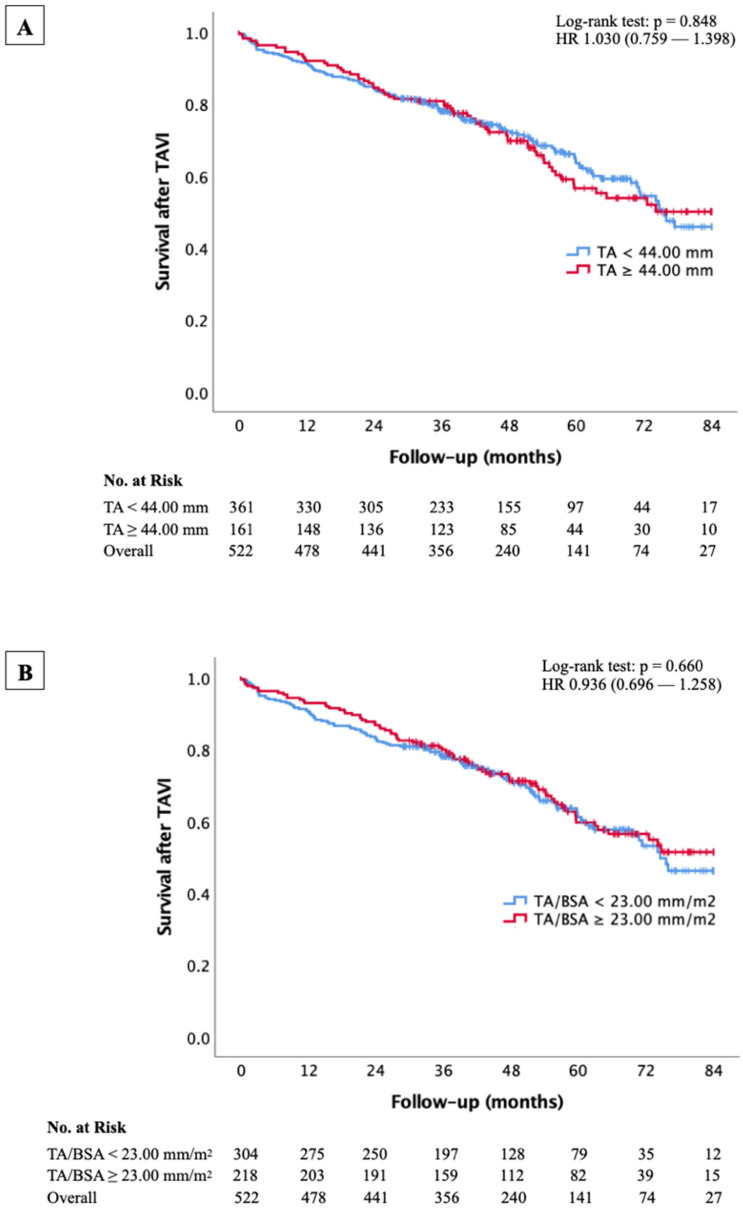
Kaplan–Meier curve with corresponding numbers at risk and log-rank tests for detection of overall mortality in dependence of different TA (**A**) and TA/BSA (**B**) cut-off values.

The Kaplan–Meier survival curves in Figure 3 illustrate the impact of TA and TA/BSA on long-term mortality in the study cohort.

Figure 3A evaluates survival based on TA cut-off values of 44.00 mm. The number at risk decreases over time, but no significant survival differences were observed between groups. The log-rank test yielded a *p*-value of 0.848, respectively, indicating no statistically significant differences in survival. Cox regression analysis confirmed these findings, with hazard ratios (HR) of 1.030 (95% CI: 0.759–1.398) for TA ≥ 44.00 mm, suggesting that annular size alone did not predict mortality.

Figure 3B analyzes survival using TA/BSA cut-offs of 23.00 mm/m^2^. Similarly to the absolute TA measurements, the determined cut-off showed no significant effect on survival, with log-rank test *p*-values of 0.660, respectively. The corresponding HR was 0.936 (95% CI: 0.696–1.258) for TA/BSA ≥ 23.00 mm/m^2^.

### 3.7. Prognostic Value of TA Measurements for Mid- and Long-Term Mortality in the TAVI Cohort

Table 4 presents the AUC values for TA and TA/BSA in predicting mid- and long-term mortality following TAVI. Across all time points, from 12-month to 84-month mortality, AUC values remain close to 0.50, indicating a lack of significant discriminatory power for TA and TA/BSA in predicting mortality. The highest AUC for TA is observed at 60 months (0.520), while for TA/BSA, the highest is at 48 months (0.501), both within a range suggesting no meaningful prognostic value. These findings suggest that TA dimensions do not serve as reliable predictors of long-term survival in this TAVI cohort.

### 3.8. Multivariate Cox Regression Analysis of Mortality Predictors

To further investigate the prognostic relevance of TA dimensions, multivariate Cox regression models regarding overall survival were constructed for both absolute TA (≥44 mm) and indexed TA/BSA (≥23.00 mm/m^2^) cut-offs (Table 5 and Table 6).

In both models, TA and TA/BSA dimensions were not significantly associated with long-term mortality. The hazard ratio (HR) for TA ≥ 44 mm was 0.907 (95% CI: 0.649–1.268, *p* = 0.569), and for TA/BSA ≥ 23 mm/m^2^, HR was 0.906 (95% CI: 0.649–1.266, *p* = 0.564), reinforcing the findings from the Kaplan–Meier and univariate Cox regression analyses.

Atrial fibrillation (AF) emerged as a strong independent predictor of mortality, with HRs of 1.890 (95% CI: 1.383–2.584, *p* < 0.001) in the TA model and 1.877 (95% CI: 1.367–2.578, *p* < 0.001) in the TA/BSA model. Other variables, such as age, LVEF, tricuspid regurgitation severity, and elevated systolic pulmonary artery pressure (sPAP ≥ 40 mmHg), did not demonstrate significant associations with mortality in either model.

## 4. Discussion

### 4.1. Reassessing the Prognostic Value of TA Dimensions in TAVI Patients

Our study provides evidence that TA dimensions, particularly TA and TA/BSA, serve as reliable prognostic indicators for long-term survival following TAVI. This finding carries important clinical implications, as it directly challenges the utility of these anatomical parameters in pre-procedural risk stratification. Despite their accessibility and reproducibility, static annular measurements appear insufficient as standalone predictors of mortality. This comprehensive analysis found no significant association between TAD and long-term mortality, contradicting previous research and necessitating a reevaluation of its clinical relevance.

### 4.2. Reassessment of Previous Findings

The work of Deseive et al. [14], which suggested a prognostic role for TAD, reported a concordance index (=AUC) of only 0.590 for TA/BSA ≥ 23.00 mm/m^2^ as a predictor of all-cause mortality in TAVI. Despite applying the same cut-off values in a comparably cohort with very similar AUC values, our analysis revealed no significant survival differences. This discrepancy raises important methodological and conceptual considerations.

Patient selection and cohort characteristics may partially explain the divergent outcomes. Deseive et al. [14] included a somewhat younger population, with a mean age of 81.7 ± 7.8 years, compared to our cohort where over 80% of patients in the TA/BSA ≥ 23.00 mm/m^2^ group were older than 80 years (83.2 ± 4.5 years). This age difference could influence the hemodynamic profile and right heart remodeling, potentially making TA enlargement a more dynamic marker in younger, less frail patients, and a less informative one in a very elderly cohort where right heart adaptation may already have plateaued.

Furthermore, in both analyses, AF had a significant impact regarding mortality prediction. In our multivariate analysis, AF (HR 1.88, *p* < 0.001) emerged as the strongest independent predictor, overshadowing the prognostic role of other echocardiogrphic and CT-morphological criteria of RV dysfunction. Interestingly, in Deseive’s model, despite AF being significant (HR 1.78, *p* < 0.001), TA/BSA still retained statistical significance. This divergence raises the possibility that in their population, annular enlargement may be acting as a surrogate for atrial or right heart dysfunction not fully captured by other variables.

In this context, it is plausible that TA/BSA reflects chronic remodeling rather than acute prognostic risk, especially in elderly patients with extensive cardiovascular comorbidities. As such, the prognostic utility of TA/BSA may be limited to specific subgroups or stages of right heart involvement, and not universally applicable.

### 4.3. Alignment with Recent Literature

Our findings align with those of Hirasawa et al. [21], who also concluded that septolateral TA/BSA was not predictive of mortality in TAVI patients (univariate cox regression: HR 1.027; 95%CI 0.982–1.073; *p* = 0.251). Their use of whole-beat CT imaging highlighted the importance of annular shape over size in determining long-term prognosis. This consistency across studies adds weight to the argument against TAD as a reliable mortality risk indicator.

### 4.4. Shifting Focus: From Anatomy to Function

Traditionally, the assessment of the right heart in TAVI patients has relied heavily on static anatomical measurements, such as RV size and tricuspid valve morphology. However, recent studies by Fortuni et al. [22] and Sun et al. [23] underscore the growing recognition that functional parameters—such as RV contractility, longitudinal strain, and the severity of TR—are critical determinants of patient outcomes. This paradigm shift acknowledges that static measurements alone may not fully capture the dynamic interplay between the RV and the tricuspid valve, particularly in the context of pressure and volume overload states that often accompany AS. By incorporating functional assessments, clinicians can achieve a more comprehensive evaluation of right heart performance, which is essential for refining risk stratification and optimizing patient selection for TAVI.

### 4.5. Methodological Considerations and Future Directions

The differences between our findings and those of Deseive et al. [14] highlight the importance of standardized methodologies in assessing TA dimensions. While CT provides excellent anatomical detail, it does not offer the functional insights available through echocardiography or cardiac MRI. Future studies may benefit from a multimodal imaging approach to better integrate structural and functional aspects of right heart pathophysiology.

### 4.6. Implications for Clinical Practice

Our study suggests that TA or TA/BSA may have limited prognostic value for long-term mortality in TAVI patients. The absence of a strong prognostic signal across various cut-off values indicates that TAD should be interpreted with caution in risk stratification. Further research is needed to determine its role within a broader clinical context.

### 4.7. Future Perspectives

The contradictory findings in the literature highlight the complex nature of risk prediction in TAVI patients. Moving forward, research should focus on the following:

1. Developing integrative models that incorporate both anatomical and functional parameters of right heart function;
2. Investigating the dynamic interplay between RV performance, TR severity, and pulmonary hemodynamics;
3. Exploring novel imaging techniques and biomarkers that may offer superior prognostic value.

By shifting away from simplistic anatomical thresholds towards more comprehensive assessments of right heart physiology, we can enhance our ability to predict outcomes and tailor treatment strategies for TAVI patients.

## 5. Limitations

This study has several limitations that should be considered when interpreting the results. The retrospective and single cohort design inherently limits the ability to establish causality between TA dimensions and long-term outcomes following TAVI. The observational nature of the study may have introduced selection bias, as the cohort was derived from a single-center, which may limit the generalizability of the findings to other populations or clinical settings. The reliance on a single imaging modality (CT) to measure TA and TA/BSA may not fully capture the dynamic and complex nature of right ventricular function and tricuspid valve pathology. Although CT provides high-resolution, three-dimensional imaging, it does not evaluate the functional aspects of the tricuspid valve or the right ventricle as comprehensively as echocardiography or magnetic resonance imaging (MRI). Furthermore, TR and RV dysfunction were assessed indirectly, primarily using echocardiographic parameters, which have inherent limitations in accuracy and reproducibility.

## 6. Conclusions

In this study, we aimed to assess the prognostic value of TA dimensions, specifically TA and TA/BSA, in predicting long-term survival following TAVI. Despite the clinical relevance of TAD in the context of right ventricular dysfunction, our findings demonstrate that TA and TA/BSA measurements do not offer meaningful prognostic value for mid- or long-term mortality in TAVI patients. The cut-off values derived for TA diameter (44.50 mm) and TA/BSA (23.00 mm/m^2^) were not significantly associated with survival outcomes, challenging the predictive potential of these parameters.

## Figures and Tables

**Table 1:** Baseline characteristics of overall cohort.

Total Cohort	
**No. (%)**	
Total	522 (100.0)
Sex (male)	260 (49.8)
Age ≤80 >80	149 (28.5) 373 (71.5)
NYHA ≥ III	210 (40.2)
Diabetes Mellitus	141 (27.0)
Arterial Hypertension	453 (86.8)
CHD	264 (50.6)
AF	189 (36.2)
PAOD	44 (8.4)
COPD	59 (11.3)
MI—Prehistory	37 (7.1)
Stroke—Prehistory	39 (7.5)
TRVmax ≥ 2.8 m/s	238 (45.6)
sPAP ≥ 40 mmHg	230 (44.1)
TAPSE/sPAP < 0.55 mmHg	229 (43.9)
**Mean ± SD**	
Age (years)	82.1 ± 5.2
Height (cm)	167.8 ± 8.8
Weight (kg)	72.9 ± 14.6
BMI (kg/m^2^)	25.8 ± 4.4
BSA (m^2^)	1.8 ± 0.2
LVEF (%)	53.0 ± 9.8
LVEDD (mm)	46.4 ± 6.8
IVSd (mm)	13.4 ± 2.1
AV Vmax (m/s)	4.4 ± 0.6
AV MPG (mmHg)	45.7 ± 12.2
TRVmax (m/s)	2.7 ± 0.8
sPAP (mmHg)	38.6 ± 17.0
TAPSE (mm)	21.9 ± 4.7
TAPSE/sPAP (mm/mmHg)	0.8 ± 0.8
TA (mm)	40.4 ± 6.7
TA/BSA (mm/m^2^)	22.4 ± 4.2
MR ≥ moderate/severe	178 (34.1)
TR ≥ moderate/severe	138 (26.4)
**Median ± IQR**	
Creatinine (mg/dL)	1.1 ± 0.5
HK (%)	38.0 ± 6.8
HB (g/dL)	12.8 ± 2.0
CK (U/L)	80.5 ± 60.3
proBNP (pg/mL)	2038.0 ± 3453.5

**Table 2:** Baseline characteristics of TA ≥ 44.00 mm and TA/BSA ≥ 23.00 mm/m^2^.

	TA < 44.00 mm	TA ≥ 44.0 mm	*p*	TA/BSA < 23.00 mm/m^2^	TA/BSA ≥ 23.00 mm/m^2^	*p*
**No. (%)**						
Total	361 (69.2)	161 (30.8)	-	307 (58.8)	213 (41.2)	-
Sex (male)	154 (42.7)	106 (65.8)	<0.001	161 (52.4)	95 (44.6)	0.041
Age ≤80 >80	106 (29.4) 255 (70.6)	43 (26.7) 118 (73.3)	0.535	107 (34.9) 200 (65.1)	41 (19.2) 172 (80.8)	<0.001
NYHA ≥ III	119 (33.0)	59 (36.6)	0.288	94 (30.6)	80 (37.5)	0.094
Diabetes Mellitus	109 (30.2)	32 (19.9)	0.014	90 (29.3)	45 (21.1)	0.024
Arterial Hypertension	316 (87.5)	137 (85.1)	0.551	259 (84.4)	183 (85.9)	0.983
CHD	181 (50.1)	83 (51.6)	0.765	149 (48.5)	109 (51.2)	0.741
AF	122 (33.8)	67 (41.6)	0.086	110 (35.8)	76 (35.7)	0.814
PAOD	32 (8.9)	12 (7.5)	0.592	31 (10.1)	13 (6.1)	0.091
COPD	44 (12.2)	15 (9.3)	0.339	38 (12.4)	18 (8.5)	0.131
MI—Prehistory	29 (8.0)	8 (5.0)	0.208	24 (7.8)	11 (5.2)	0.209
Stroke—Prehistory	28 (7.8)	11 (6.8)	0.711	24 (7.8)	14 (6.6)	0.542
TRVmax ≥ 2.8 m/s	147 (40.7)	91 (56.5)	0.001	122 (39.7)	115 (54.0)	0.003
sPAP ≥ 40 mmHg	138 (38.2)	92 (57.1)	<0.001	112 (36.5)	115 (54.0)	<0.001
TAPSE/sPAP < 0.55 mmHg	143 (39.6)	86 (53.4)	0.004	109 (35.5)	118 (55.4)	<0.001
**Mean ± SD**						
Age (years)	81.8 ± 5.4	82.6 ± 4.8	0.090	81.2 ± 5.6	83.2 ± 4.5	<0.001
Height (cm)	166.9 ± 8.7	170.0 ± 8.6	<0.001	169.3 ± 8.4	165.7 ± 8.9	<0.001
Weight (kg)	72.5 ± 15.0	73.9 ± 13.9	0.320	77.3 ± 14.8	66.6 ± 11.9	<0.001
BMI (kg/m^2^)	25.9 ± 4.6	25.4 ± 4.0	0.221	26.9 ± 4.6	24.2 ± 3.6	<0.001
BSA (m^2^)	1.8 ± 0.2	1.8 ± 0.2	0.037	1.9 ± 0.2	1.7 ± 0.2	<0.001
LVEF (%)	53.6 ± 9.3	51.8 ± 10.8	0.069	53.6 ± 9.6	52.2 ± 10.1	0.125
LVEDD (mm)	45.8 ± 6.7	47.6 ± 6.7	0.012	46.5 ± 6.8	46.2 ± 0.7	0.641
IVSd (mm)	13.4 ± 2.1	13.4 ± 2.0	0.952	13.5 ± 2.1	13.3 ± 2.0	0.421
AV Vmax (m/s)	4.4 ± 0.6	4.4 ± 0.5	0.793	4.4 ± 0.6	4.3 ± 0.5	0.436
AV MPG (mmHg)	45.7 ± 12.3	45.6 ± 12.0	0.932	46.0 ± 12.6	45.0 ± 11.5	0.372
TRVmax (m/s)	2.6 ± 0.9	2.9 ± 0.7	<0.001	2.6 ± 0.9	2.8 ± 0.7	<0.001
sPAP (mmHg)	36.4 ± 16.7	43.3 ± 16.8	<0.001	36.1 ± 17.1	42.7 ± 16.1	<0.001
TAPSE (mm)	21.8 ± 4.6	22.1 ± 5.0	0.534	22.0 ± 4.4	21.5 ± 5.1	0.220
TAPSE/sPAP (mm/mmHg)	0.9 ± 0.8	0.7 ± 0.6	0.003	0.9 ± 0.8	0.6 ± 0.5	<0.001
TA (mm)	36.9 ± 4.4	48.2 ± 3.7	<0.001	36.7 ± 4.9	45.6 ± 5.3	<0.001
TA/BSA (mm/m^2^)	20.6 ± 3.2	26.4 ± 3.3	<0.001	19.6 ± 2.3	26.4 ± 2.7	<0.001
**Median ± IQR**						
Creatinine (mg/dL)	1.1 ± 0.5	1.0 ± 0.5	0.816	1.1 ± 0.5	1.0 ± 0.5	0.955
HK (%)	37.4 ± 7.0	38.4 ± 7.3	0.480	38.2 ± 6.9	37.8 ± 7.0	0.954
HB (g/dL)	12.7 ± 2.0	13.1 ± 2.1	0.280	12.7 ± 2.1	12.9 ± 2.2	0.741
CK (U/l)	84.3 ± 60.0	77.5 ± 63.3	0.600	77.5 ± 62.0	84.5 ± 62.8	0.603
proBNP (pg/mL)	1947.0 ± 3868.0	2170.0 ± 2903.8	0.571	2152.0 ± 3891.6	1807.0 ± 3024.3	0.202

**Table 3:** Procedural outcome data of short-term mortality regarding TA and TA/BSA.

	TA < 44.00 mm	TA ≥ 44.0 mm	*p*	TA/BSA < 23.00 mm/m^2^	TA/BSA ≥ 23.00 mm/m^2^	*p*
**No. (%)**						
Procedural death	2 (0.6)	1 (0.6)	0.925	2 (0.7)	1 (0.5)	0.772
30-day death	4 (1.1)	3 (1.9)	0.488	3 (1.0)	4 (1.9)	0.399
30-day stroke/TIA	9 (2.5)	3 (1.9)	0.657	6 (2.0)	6 (2.8)	0.790
30-day pacemaker necessity	46 (12.7)	20 (12.4)	0.919	40 (13.0)	26 (12.2)	0.590
30-day major vascular complications	16 (4.4)	6 (3.7)	0.717	12 (3.9)	10 (4.7)	0.900

**Table 4:** AUC values of mid- and longterm mortality regarding TA and TA/BSA.

AUC (95% CI)	TA	TA/BSA
12-month-mortality	0.503 (0.419–0.587)	0.457 (0.373–0.542)
24-month-mortality	0.486 (0.420–0.553)	0.465 (0.399–0.532)
36-month-mortality	0.478 (0.419–0.538)	0.474 (0.413–0.534)
48-month-mortality	0.518 (0.461–0.574)	0.501 (0.445–0.557)
60-month-mortality	0.520 (0.466–0.574)	0.508 (0.454–0.562)
72-month-mortality	0.516 (0.463–0.569)	0.504 (0.451–0.557)
84-month-mortality	0.515 (0.462–0.568)	0.498 (0.446–0.551)

**Table 5:** Multivariate Cox regression model of long-term mortality regarding TA.

Cox Regression	HR (95% CI)	*p*
TA ≥ 44.00 mm	0.907 (0.649–1.268)	0.569
Age (per 1 year)	1.022 (0.990–1.055)	0.178
LVEF (per 1%)	0.992 (0.976–1.009)	0.352
AF	1.890 (1.383–2.584)	<0.001
TR ≥ moderate/severe	0.823 (0.561–1.208)	0.319
sPAP ≥ 40 mmHg	1.311 (0.933–1.843)	0.118

**Table 6:** Multivariate Cox regression model of long-term mortality regarding TA/BSA.

Cox Regression	HR (95% CI)	*p*
TA/BSA ≥ 23.00 mm/m^2^	0.906 (0.649–1.266)	0.564
Age (per 1 year)	1.025 (0.992–1.058)	0.140
LVEF (per 1%)	0.993 (0.977–1.010)	0.445
AF	1.877 (1.367–2.578)	<0.001
TR ≥ moderate/severe	0.840 (0.563–1.254)	0.394
sPAP ≥ 40 mmHg	1.295 (0.915–1.831)	0.145

## Data Availability

The original contributions presented in this study are included in the article. Further inquiries can be directed to the corresponding authors.
